# Prevalence of and Factors Associated with Albuminuria in the Korean Adult Population: The 2011 Korea National Health and Nutrition Examination Survey

**DOI:** 10.1371/journal.pone.0083273

**Published:** 2013-12-27

**Authors:** Jong Chul Won, Yun Jeong Lee, Jung Min Kim, Sang Youb Han, Jung Hyun Noh, Kyung Soo Ko, Byoung Doo Rhee, Dong-Jun Kim

**Affiliations:** 1 Department of Internal Medicine, Sanggye Paik Hospital, Cardiovascular and Metabolic Disease Center, College of Medicine, Inje University, Seoul, Republic of Korea; 2 Department of Internal Medicine, Ilsan-Paik Hospital, College of Medicine, Inje University, Koyang, Gyeonggi-do, Republic of Korea; University of Glasgow, United Kingdom

## Abstract

**Background:**

Microalbuminuria is associated with increased risk of renal disease and cardiovascular diseases even in non-diabetic subjects. High incidence rates of microalbuminuria have been found in a number of population-based studies. However, the prevalence and risk factors associated with microalbuminuria in the general population in Korea are unclear.

**Objectives:**

The present study was performed to estimate the prevalence of microalbuminuria and investigate the associated risk factors in the general adult population using the Fifth Korea National Health and Nutrition Examination Survey (KNHANES V-2) data from 2011.

**Methods:**

A total of 5,202 participants (mean age, 45.6 years; men, 2,337; women, 2,865) were included in the analysis. Microalbuminuria was evaluated in participants of KNHANES V-2 based on the urine albumin–creatinine ratio. Estimated glomerular filtration rate was calculated using the Modification of Diet in Renal Disease study equation.

**Results:**

The weighted prevalence of microalbuminuria was 5.2% (95% CI, 4.4–6.1) in the general population. The prevalence of albuminuria is increased with age. After adjustment for age and sex, the presence of albuminuria was associated with increased waist circumference, systolic and diastolic blood pressure, aspartate aminotransferase, triglyceride, fasting plasma glucose, and the presence of hypertension and diabetes. In logistic regression analyses, older age, female sex, diabetes, hypertension, and serum aspartate aminotransferase were independently associated with the presence of albuminuria.

**Conclusion:**

The prevalence of microalbuminuria was found to be 5.2%, and conventional risk factors for cardiovascular diseases are closely related to the presence of microalbuminuria in Korea. Microalbuminuria may be a useful marker to identify individuals with increased risk of cardiovascular disease.

## Introduction

In 2009, the overall prevalence of end-stage renal disease (ESRD) in Korea was reported to be 1,113.6 per million subjects, and the prevalence rate increased by about 12% per year during 2000–2009. The high prevalence of diabetes and the associated renal complications have been described for these years in Korea [1]. Diabetic nephropathy is the most common cause of ESRD (45.4% in new patients with ESRD) [2].

Microalbuminuria is an important marker of progression to ESRD as well as increased risk of cardiovascular disease (CVD) and mortality across all levels of glomerular filtration rate (GFR) [3], [4]. Given these well-established links, there is concern regarding the high prevalence of microalbuminuria in a number of populations worldwide because effective interventions are needed to manage risk factors associated with microalbuminuria. These studies showed that the prevalence of microalbuminuria is increased in subjects with one or more CVD risk factors, such as elevated blood pressure or high triglyceride (TG) concentration and low high-density lipoprotein cholesterol (LDL-C) concentration, indicating that microalbuminuria is an early marker of systemic endothelial dysfunction and may be an important clinical sign of adverse health outcomes even in the general adult population [5]. Therefore, it is necessary to estimate its prevalence and risk factors to prepare effective strategies to attenuate the impact of systemic vascular disease on health care costs and quality of life due to substantial renal disease, particularly given the increases in prevalence of obesity, diabetes, and hypertension in Korea [1], [6]. However, little information is available to estimate the prevalence of albuminuria and associated risk factors in the Korean population. This study was performed to investigate these issues in the general population using the fifth Korea National Health and Nutrition Examination Survey (KNHANES V-2) data from 2011.

## Materials and Methods

### Study population and data collection

This study used data from KNHANES V-2, a national probability survey conducted by the Korean Center for Disease Control for Health Statistics at 192 survey locations to determine the health and nutritional status of the civilian, non-institutionalized Korean population since 1998. The Korean Center for Disease Control conducted a series of KNHANES, in 1998, 2001, 2005, 2007–2009, and 2010–2012. In KNHANES V-2, an annual total of 3,840 households were selected, and urine albumin was first measured starting in 2011. A standardized interview was conducted in the homes of the participants to collect information on demographic variables, family history, medical history, medications used, and a variety of other health-related variables. Participants were chosen using proportional allocation-systemic sampling with multistage stratification (age, sex, and region). This study was approved by the institutional review board of Ilsan Paik Hospital, Republic of Korea (IB-1308-031). After approval of the study proposal, the KNHANES dataset was made available at the request of the investigator. As the dataset did not include any personal information and participants' consent had already been given for the KNHANES, our study was exempt from participant consent.

### Health interview, examination, and laboratory tests

The Health Interview included well-established questions to determine the demographic and socioeconomic characteristics of the subjects including questions on age, education level, occupation, income, marital status, smoking habit, alcohol consumption, exercise, previous and current diseases, and family disease history. Smoking status was divided into three categories: current smoker, ex-smoker, and nonsmoker. Subjects were questioned about whether they exercised with an intensity that left them with slight difficulty in breathing and sweating. Subjects who exercised regularly at a moderate intensity were asked about the frequency at which they exercised per week and the length of time per exercise session. Regular exercise was defined exercising as five or more times per week. Alcohol consumption was assessed by questioning the subjects about their drinking behavior during the month before the interview. Heavy alcohol drinking was categorized as drinking four or more times per week.

Height and weight were obtained using standardized techniques and equipment. Height was measured to the nearest 0.1 cm using a portable standiometer (Seriter, Bismarck, ND). Weight was measured to the nearest 0.1 kg using a Giant-150N calibrated balance-beam scale (Hana, Seoul, Korea). Body mass index (BMI) was calculated by dividing weight by the square of height (kg/m^2^). Systolic and diastolic blood pressure (BP) was measured by standard methods using a sphygmomanometer with the patient in the sitting position. Three measurements were made for all subjects at 5-min intervals; the average of the second and third measurements was used in the analysis. Blood samples were collected in the morning after fasting for at least 8 h and single-spot urine specimen collected in the first morning void. Analysis of fasting plasma glucose and serum total cholesterol, TG, LDL-C and high-density lipoprotein cholesterol levels were performed by Hitachi Automatic Analyzer 7600 (Hitachi, Tokyo, Japan). Serum gamma-glutamyl transpeptidase (GGT) was measured using an enzymatic colorimetric method and aspartate aminotransferase (AST) and alanine transaminase (ALT) were measured using the standardized kinetic method (Modular P, Roche Diagnostics, Indianapolis, IN). Obesity was defined as BMI ≥25 kg/m^2^ [7]. Hypertension was defined as systolic BP ≥140 mmHg, diastolic BP ≥90 mmHg, or use of antihypertensive medications irrespective of BP. Diabetes was defined as fasting plasma glucose ≥7.0 mmol/l, current anti-diabetes medication, or a previous diagnosis of diabetes by a doctor.

### Assessment and definition of microalbuminuria and estimation of glomerular filtration rate (eGFR)

Urine albumin and creatinine concentrations were measured in the same laboratory during all surveys. Serum and urinary concentrations of creatinine were measured using a colorimetric method (Hitachi Automatic Analyzer 7600, Hitachi, Tokyo, Japan). The inter-assay coefficient of variation for serum creatinine was <1.4%. Urinary albumin was measured in random urine samples using a turbidimetric immunoassay (Hitachi Automatic Analyzer 7600; Hitachi). Laboratory control measures in KNHANES V-2 indicated that these assays are highly reliable with consistently low coefficients of variation (3.1%). The ratio of urinary albumin to urinary creatinine was reported as albumin-creatinine ratio (ACR) in milligrams per gram of creatinine. Subjects were divided according to albuminuria status, i.e., normoalbuminuria (ACR <20 mg/g Cr), microalbuminuria (ACR = 30–299 mg/g Cr), or macroalbuminuria (ACR≥300 mg/g Cr) [8]. eGFR was calculated using the abbreviated equation from the Modification of Diet in Renal Disease (MDRD) study: eGFR (ml/min/1.73 m^2^) = 175×(S_Cr_/88.4, µmol/l)^−1.154^×Age^−0.203^×0.742 (if female) [9]. Additionally, subjects were stratified by eGFR value (ml/min/1.73 m^2^) into four groups: group 1, eGFR ≥90 (*n* = 1988); group 2, eGFR 60–89 (*n* = 2981); group 3, eGFR 30–59 (*n* = 215); group 4, eGFR <30 ml/min/1.73 m^2^ (*n* = 8) [10]. Decreased eGFR was defined as <60 ml/min/1.73 m^2^ in this study.

### Statistical analyses

Data are presented as mean and 95% confidence interval (CI). Participants in Korean NHANES were not sampled randomly. Korean NHANES was designed as a complex, stratified, multistage probability-sampling model. Therefore each participant does not have the same power for representation of whole Korean population. If we try to present prevalence in whole Korean population form the dataset, we should consider how much power each participant for representation (sample weight) for whole Korean population. After approval of investigator's proposal by the Korean Center for Disease Control, the Korean Center for Disease Control provide survey dataset including information about survey location, strata by age, sex, and other factors, and sample weight of each participants to investigator. Survey sample weights, calculated taking the sampling rate, response rate, and age/sex proportion of the reference population (2005 Korean National Census Registry) into consideration, were used in all analyses to produce estimates representative of the non-institutionalized Korean civilian population. Age and age-adjusted comparisons of clinical characteristics according to the presence of microalbuminuria or macroalbuminuria were performed using analysis of covariance (ANCOVA). To determine which parameters were associated with the presence of microalbuminuria or macroalbuminuria, logistic regression analysis was conducted with age, sex, waist circumference, presence of hypertension, serum AST, serum TG, impaired fasting glucose, and diabetes as variables. Linear regression analysis for log ACR was performed with age, sex, waist circumference, systolic BP, fasting plasma glucose, serum TG, and serum AST as a continuous variable. Two-tailed analyses were conducted, and *P*<0.05 was deemed to indicate statistical significance. Statistical analyses were performed using SPSS software (ver. 18.0 for Windows; SPSS, Chicago, IL).

## Results

### Study population

A total of 10,589 people participated in the KNHANES V-2, 2011. The participation rate for the health examination (including laboratory tests) was 76.1%. Of these, the 5,202 participants aged over 19 years who completed the measurement of ACR were included in the analysis. Sample data were weighted appropriately to account for the complex design and unequal probability of selection of sample subjects to compute prevalence estimates of abnormal albumin excretion levels for the Korean population.

### Demographic and clinical characteristics of the study population

Table 1 shows the weighted demographic and clinical characteristics of the study population entered into the final analysis. The overall mean age was 45.6 years old (95% CI, 44.7–46.5). Both sexes were equally represented. The weighted mean age was 44.2 years (43.2–45.2) for men and 47.2 years (46.2–48.2) for women. The weighted mean BMI was 23.8 kg/m^2^ (95% CI, 23.6–23.9), serum creatinine level (S_Cr_) was 79.6 µmol/l (95% CI, 70.7–79.6), and median ACR was 2.34 mg/g Cr (inter-quartile, 0.84–6.61). And, 33% of subjects were obese and 9% had diabetes. Twenty-seven percent of subjects was hypertension: systolic BP≥140 or diastolic BP≥90 mmHg without antihypertensive medication (10.8%, 563/5202), or use of antihypertensive medications irrespective of BP (21.6%, 1124/5202).

**Table 1:** Weighted demographic and clinical characteristics of the Korean population ≥19 years old in the 2011 KNHANES.

*N*, (unweighted/weighted)	5,202/32,333,446
Age (years)	45.6 (44.7–46.5)
Men (%)	52.3 (50.9–53.8)
Current smoking (%)	27.3 (25.5–29.1)
Heavy alcohol drinking (%)	11.7 (6.8–8.7)
Regular exercise (%)	13.2 (11.8–14.7)
WC (cm)	81.7 (81.2–82.1)
BMI (kg/m^2^)	23.8 (23.6–23.9)
Obesity (%)	32.9 (30.9–34.8)
Diabetes (%)	9.0 (8.0–10.1)
Hypertension (%)	26.5 (24.8–28.2)
Systolic BP (mmHg)	117.7 (116.0–118.5)
Diastolic BP (mmHg)	76.3 (75.9–76.8)
Serum Creatinine (µmol/l)	79.6 (70.7–79.6)
eGFR, ml/min/1.73 m^2^	89.1 (88.3–89.8)
ACR (mg/g Cr)	2.34 (0.84–6.61)
FPG (mmol/l)	5.4 (5.3–5.4)
Serum LDL-C (mmol/l)	2.9 (2.9–3.0)
Serum TG (mmol/l)	1.5 (1.5–1.6)
AST (U/l)	22.5 (22.1–22.9)
ALT (U/l)	22.2 (21.5–22.9)
GGT (U/l)	35.4 (33.6–37.3)
Anti-hypertensive drug (%)	14.9 (13.7–16.3)
Anti-lipid drug (%)	4.4 (3.8–5.0)

Data are expressed as means with 95% confidence intervals except for ACR expressed as median with inter-quartile range. WC, waist circumference; BMI, body mass index; BP, blood pressure; eGFR, estimated glomerular filtration rate; ACR, albumin–creatinine ratio; FPG, fasting plasma glucose; LDL-C, low-density lipoprotein cholesterol; TG, triglyceride; AST, aspartate aminotransferase; ALT, alanine aminotransferase; GGT, γ-glutamyltransferase.

### Prevalence of microalbuminuria by age, sex, and eGFR

**Figure 1:**
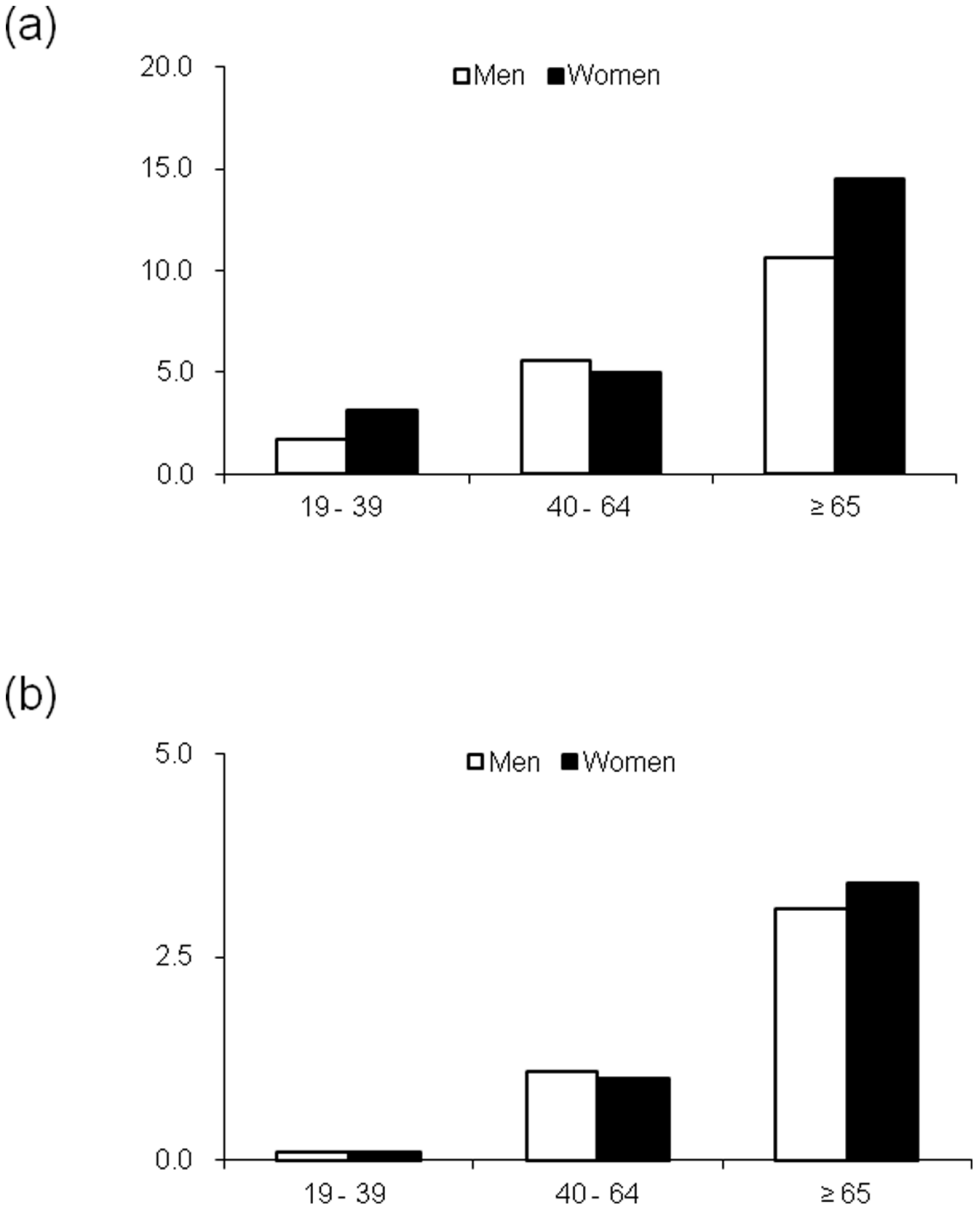
Weighted prevalence (%) of microalbuminuria (a) and macroalbuminuria (b) by age (years) in the Korean population ≥19 years old in the 2011 KNHANES. Weighted *n*: men 19–39 years, 7,985,505; 40–64 years, 7,946,568; and ≥65 years, 1,873,929; women, 5,458,591, 7,426,414, and 2,529,438, respectively.

Table 2 shows the weighted prevalence rates of microalbuminuria and macroalbuminuria according to age, sex, and eGFR. The weighted prevalence rates of microalbuminuria and macroalbuminuria were 5.2% (95% CI, 4.4–6.1) and 1.0% (0.7–1.4), respectively. These prevalence rates were significantly increased with age and were high in subjects of both sexes aged ≥65 years (*P*<0.001). Generally, the prevalence of microalbuminuria or macroalbuminuria was not different according to sex. In subjects <65 years old, the prevalence was higher in men. The reverse pattern was observed in subjects ≥65 years old, but the difference was not statistically significant (Figure 1).

**Table 2:** Weighted prevalence of albuminuria by age, sex, and eGFR of the Korean population ≥19 years old in the 2011 KNHANES.

	Normoalbuminuria % (95% CI)	Microalbuminuria, % (95% CI)	Macroalbuminuria % (95% CI)	
Total	93.8 (92.9–94.7)	5.2 (4.4–6.1)	1.0 (0.7–1.4)	*P*
Age, year				<0.001
19–39	97.6 (96.3–98.4)	2.3 (1.5–3.5)	0.1 (0.0–0.4)	
40–64	93.6 (92.4–94.7)	5.3 (4.4–6.5)	1.1 (0.6–1.8)	
≥65	83.9 (81.0–86.5)	12.8 (10.8–15.2)	3.2 (2.1–5.0)	
Sex				0.114
Men	94.5 (93.3–95.5)	4.5 (3.6–5.7)	0.9 (0.6–1.5)	
Women	93.1 (91.8–94.1)	5.9 (4.9–7.1)	1.1 (0.7–1.6)	
eGFR				<0.001
≥90	96.1 (94.9–96.9)	3.5 (2.7–4.6)	0.4 (0.2–0.8)	
60–89	93.1 (91.8–94.2)	5.8 (4.8–6.9)	1.1 (0.7–1.7)	
30–59	73.9 (64.7–81.4)	19.0 (12.5–27.9)	7.1 (4.2–11.8)	
<30	33.9 (6.5–79.0)	27.8 (7.3–65.3)	38.3 (12.9–72.2)	

Data are expressed as estimated prevalence (%) and 95% confidence intervals. eGFR, estimated glomerular filtration rate (ml/min/1.73 m^2^).

The prevalence of decreased eGFR (<60 ml/min/1.73 m^2^) was 2.8% (95% CI, 2.4–3.3) (Table 3). When subjects with microalbuminuria or macroalbuminuria were stratified by eGFR, among subjects with eGFR of 30–59 ml/min/1.73 m^2^, the prevalence rates of microalbuminuria and macroalbuminuria were 19.0% (95% CI, 12.5–27.9) and 7.1% (4.2–11.8), respectively. The rates in cases with eGFR <30 ml/min/1.73 m^2^ were 27.8% (95% CI, 7.3–65.3) and 38.3% (12.9–72.2), respectively (Table 2). On the other hand, about 90% and 80% of subjects with microalbuminuria and macroalbuminuria, respectively, had relatively preserved eGFR (≥60 ml/min/1.73 m^2^) (Table 4).

**Table 3:** Prevalence of decreased eGFR (eGFR <60 ml/min/1.73 m^2^) by age and sex of the Korean population ≥19 years old in 2011 KNHANES.

	Unweighted prevalence %	Weighted prevalence % (95% CI)	
Total	4.3 (223/5,202)	2.8 (2.4–3.3) (911,809/32,333,446)	*P*
Age, year			<0.001
19–39	0.1	0.1 (0.0–0.8)	
40–64	2.0	2.1 (1.5–2.8)	
≥65	13.7	13.2 (10.9–15.9)	
Gender			0.518
Men	4.8	3.0 (2.3–3.7)	
Women	3.8	2.7 (2.1–3.4)	

Data are expressed as estimated prevalence (%) and 95% confidence intervals.

**Table 4:** Weighted number and distribution of eGFR in each category of albuminuria in Korean adult population.

	Normoalbuminuria		Microalbuminuria		Macroalbuminuria	
eGFR	Weighted *N*	%*	Weighted *N*	%*	Weighted *N*	%*
≥90	1,3583,888	44.8 (42.4–47.1)	500,437	29.9 (24.1–36.4)	57,698	17.9 (9.0–32.5)
60–89	1,6090,965	53.0 (50.8–55.3)	997,347	59.6 (53.2–65.7)	191,302	59.4 (44.2–73.0)
30–59	653,846	2.2 (1.7–2.7)	168,312	10.1 (6.6–15.0)	62,886	19.5 (11.5–31.3)
<30	9,067	0.1 (0.0–0.2)	7,440	0.4 (0.1–2.0)	10,257	3.2 (1.0–9.7)

*Proportion of each eGFR group. Data are expressed as estimated prevalence (%) and 95% confidence intervals. *χ* ^2^ test, *P*<0.001. eGFR, estimated glomerular filtration rate (ml/min/1.73 m^2^).

### Clinical characteristics of the adult population according to the presence of albuminuria

Table 5 summarizes age-, sex-, and age- and sex-adjusted clinical characteristics of the adult population according to the presence of microalbuminuria and macroalbuminuria. The prevalence of albuminuria increased with age. There were no differences in the presence of albuminuria by sex. After simultaneously controlling for age and sex, results showed that waist circumference, systolic and diastolic BP, AST, TG, fasting plasma glucose, and the presence of hypertension and diabetes were significantly different among three groups. However, there was no difference in current smoking, heavy alcohol drinking, regular exercise, ALT, GGT, LDL-C, or proportion of subjects taking lipid-lowering drugs.

**Table 5:** Age-, sex-, and age- and sex-adjusted demographic and clinical characteristics of the Korean population ≥19 years old in the 2011 KNHANES by categories of albuminuria.

	Normoalbuminuria	Microalbuminuria	Macroalbuminuria	*P* for trend
Number (weighted)	30,337,766	1,673,536	322,143	
Age (years)	44.8 (44.0–45.7)	57.1 (54.6–59.5)	60.4 (55.8–64.9)	<0.001
Women (%)	47 (46–49)	54 (48–61)	50 (36–65)	0.126
Current smoking (%)	27.1 (25.4–28.7)	30.8 (24.5–37.0)	27.7 (15.5–39.9)	0.510
Heavy alcohol drinking (%)	7.8 (6.8–8.8)	6.4 93.2–9.7)	7.7 (1.0–16.7)	0.740
Regular exercise (%)	13.4 (11.9–14.9)	9.0 (5.4–12.6)	14.4 (4.0–24.8)	0.079
WC (cm)	81.5 (81.0–82.0)	84.6 (82.8–86.3)	84.5 (81.8–87.2)	<0.001
Obesity (%)	32.3 (30.4–34.3)	41.0 (33.2–48.9)	38.5 (24.1–53.0)	0.066
Diabetes (%)	7.9 (6.9–8.8)	23.5 (17.5–29.6)	38.6 (24.6–52.6)	<0.001
Hypertension (%)	24.8 (23.3–26.3)	50.9 (43.4–58.4)	54.8 (41.6–68.0)	<0.001
Systolic BP (mmHg)	117.1 (116.5–117.7)	125.9 (123.3–128.5)	134.1 (128.5–139.7)	<0.001
Diastolic BP (mmHg)	76.1 (75.7–76.5)	79.5 (78.0–81.1)	80.2 (76.3–84.0)	<0.001
Serum Creatinine (µmol/l)	75.1 (74.3–76.0)	76.9(75.1–79.6)	88.4(81.3–96.4)	0.001
eGFR, ml/min/1.73 m^2^	89.1 (88.4–89.9)	88.9 (86.4–91.3)	81.2 (75.6–86.7)	0.023
ACR (mg/g Cr)	2.14 (0.77–4.66)	60.35 (40.73–114.93)	575.30 (403.95–977.42)	<0.001
FPG (mmol/l)	5.3 (5.3–5.4)	5.8 (5.6–5.9)	6.9 (6.0–7.8)	<0.001
Serum LDL-C (mmol/l)	2.9 (2.9–3.0)	3.0 (2.8–3.2)	2.9 (2.0–3.7)	0.867
Serum TG (mmol/l)	1.5 (1.5–1.6)	1.7 (1.6–1.9)	2.6 (1.8–3.4)	<0.001
AST (U/l)	22.4 (22.0–22.8)	24.8 (22.7–26.8)	25.2 (20.9–29.5)	0.023
ALT (U/l)	22.1 (21.4–22.7)	24.7 (21.8–27.6)	22.2 (18.2–26.1)	0.185
GGT (U/l)	35.1 (33.2–36.9)	41.7 (31.6–51.8)	39.3 (26.7–51.8)	0.367
Anti-hypertensive drug (%)	13.8 (12.7–15.0)	30.7 (23.8–37.7)	36.9 (22.6–51.1)	<0.001
Anti-lipid drug (%)	4.3 (3.7–5.0)	6.0 (2.7–9.2)	4.3 (1.0–10.7)	0.640

Data are expressed as means with 95% confidence intervals except for ACR expressed as median with inter-quartile range. WC, waist circumference; BMI, body mass index; BP, blood pressure; eGFR, estimated glomerular filtration rate; ACR, albumin–creatinine ratio; FPG, fasting plasma glucose; LDL-C, low-density lipoprotein cholesterol; TG, triglyceride; AST, aspartate aminotransferase; ALT, alanine aminotransferase; GGT, γ-glutamyltransferase.

### Factors associated with albuminuria

**Figure 2:**
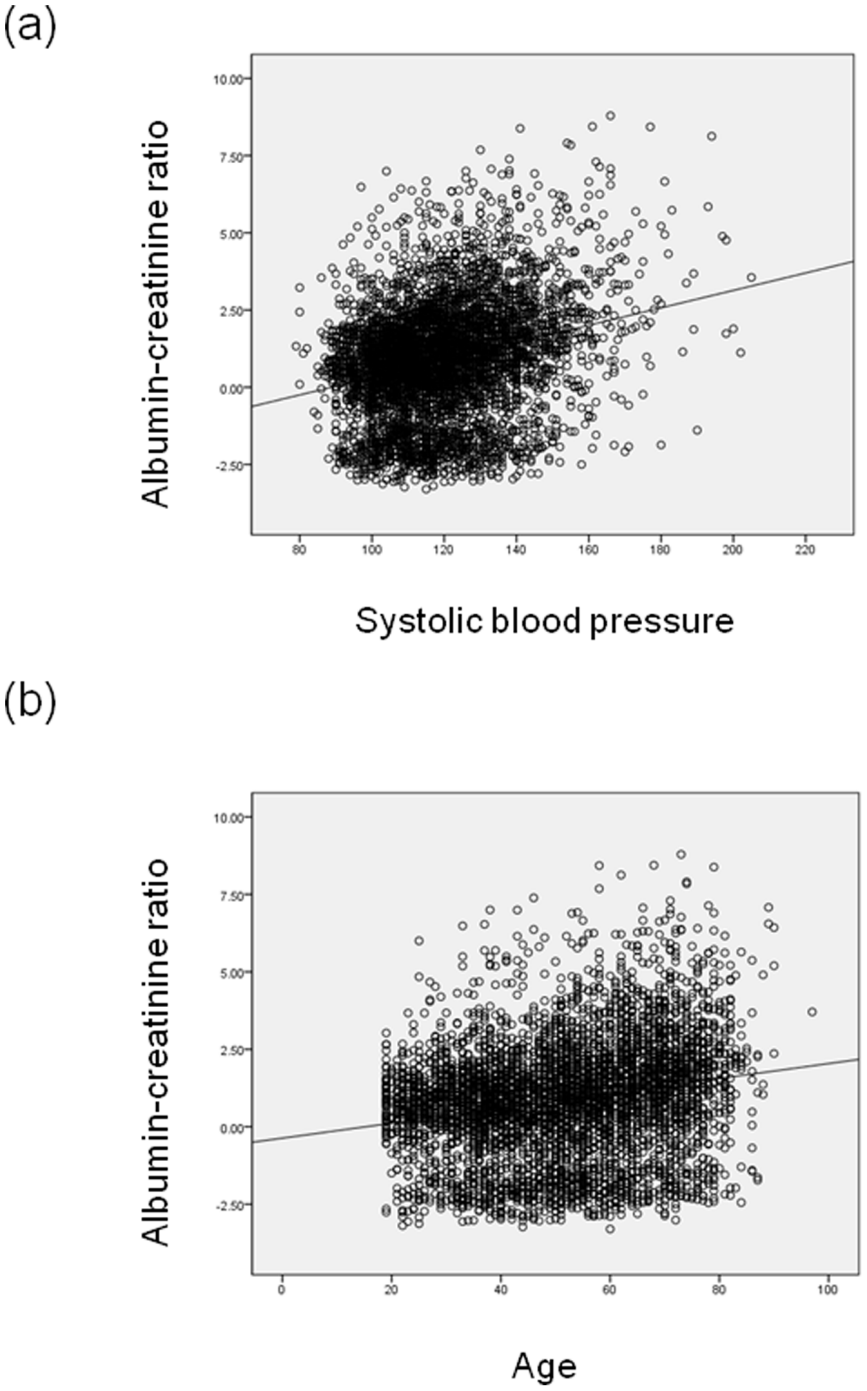
The relationship between the albumin-creatinine ratio (mg/g Cr, log transformed) versus systolic blood pressure (mmHg) (a, *r* = 0.267, *P*<0.001) and age (yr) (b, *r* = 0.214, *P*<0.001).

Multiple logistic regression analysis showed that age, female sex, AST, hypertension, and diabetes were independently associated with the presence of any albuminuria including micro- and macro-albuminuria (Table 6). Age, hypertension, TG, and diabetes were independently associated with the presence of macroalbuminuria. Linear regression analysis for log ACR was performed with age, sex, waist circumference, systolic BP, fasting plasma glucose, serum TG, and serum AST as a continuous variable (Figure 2). In this model, age, female sex, systolic BP, fasting plasma glucose, and serum AST was independently associated with log transformed ACR (Table 7).

**Table 6:** Multiple logistic regression analyses for albuminuria or macroalbuminuria.

	Albuminuria^a^		Macroalbuminuria^b^	
	OR (95% CI)	*P*	OR (95% CI)	*P*
Age (year), 19–39				
40–64	1.47 (0.95–2.27)	0.087	4.68 (1.15–19.08)	0.031
≥65	2.57 (1.57–4.22)	<0.001	9.42 (2.47–35.94)	0.001
Men	0.65 (0.49–0.85)	0.002	0.82 (0.46–1.48)	0.505
Waist circumference				
per 10 cm increase	1.17 (0.99–1.38)	0.071	1.04 (0.76–1.43)	0.791
Hypertension	3.24 (2.25–4.66)	<0.001	2.94 (1.37–6.30)	0.006
Normal fasting glucose				
IFG	1.10 (0.75–1.62)	0.613	0.57 (0.21–1.54)	0.270
Diabetes	2.85 (1.97–4.15)	<0.001	3.54 (1.82–6.87)	<0.001
Serum TG				
per 0.2 mmol/l increase	1.02 (1.00–1.04)	0.060	1.06 (1.03–1.10)	<0.001
Serum AST				
per 5 U/l increase	1.05 (1.01–1.09)	0.020	1.02 (0.91–1.14)	0.694

^a^ Microalbuminuria or macroalbuminuria versus normoalbuminuria (reference).

^b^ Macroalbuminuria versus normo-or microalbuminuria. IFG, impaired fasting glucose; TG, triglyceride; AST, aspartate aminotransferase.

**Table 7:** Linear regression analyses for albumin-creatinine ratio*.

	Coefficient (95% CI)	*P*
Age (year)	0.007 (0.004–0.011)	<0.001
Women	0.419 (0.320–0.519)	<0.001
WC (cm) (10 cm increase)	0.003 (−0.002–0.008)	0.234
Systolic BP (mmHg) ffffpppressurepressHypertension	0.021 (0.018–0.024)	<0.001
Fasting plasma glucose (mmol/l)	0.266 (0.226–0.305)	<0.001
Serum TG (mmol/l)	0.015 (−0.027–0.056)	0.494
Serum AST (U/l)	0.006 (0.001–0.010)	0.009

*Log transformed values of albumin-creatinine ratio were used. WC, waist circumference; BP, blood pressure; TG, triglyceride; AST, aspartate aminotransferase.

## Discussion

These survey data from a representative sample of the Korean population indicated a microalbuminuria prevalence rate of 5.2% (95% CI, 4.4–6.1). Older age, female sex, hypertension, serum TG, and diabetes are independently associated with the presence of albuminuria in the Korean adult population. Microalbuminuria has been shown to be an early sign of progressive renal failure and increased risk for CVD, not only in diabetic or hypertensive patients but also in non-diabetic and non-hypertensive subjects [11], [12]. As microalbuminuria reflects increased endothelial permeability and intraglomerular capillary pressure, it may be a useful clinical marker of endothelial dysfunction and vascular dysfunction even in the general population [13], [14]. Despite these clinical implications and the increased burden of diabetic and non-diabetic renal disease in Korea [1], [2], few population-based screening studies for albuminuria in Korean populations have been performed.

The prevalence of microalbuminuria in this study was slightly lower than those in Australia (6.0%) [15], Europe (7%) [11], and that in the National Health and Nutrition Examinations Survey (NHANES) III in the US (7.8%) [16]. However, our results were consistent with previous reports conducted in general populations without ethnic variation in Korea (5.4%) and in Japan (4.6%) [17], [18]. These discrepancies may have been due mainly to differences in the populations analyzed, diagnostic criteria, or method used for measurement of microalbuminuria. The prevalence of microalbuminuria increases with age. Additionally, the mean age of our subjects was 45.6 years, which was younger than that in other studies. The proportion of subjects with diabetes or hypertension could also be a potential explanation for these differences. In the present study population, the proportions of subjects with diabetes and hypertension were 9.0% and 26.5%, respectively. These are similar to the values in a previous nationwide study in the general population in Korea [1]. Additionally, urinary excretion of creatinine can vary with ethnicity, and dietary pattern (i.e., relatively low levels of animal protein in Koreans) affects the urinary excretion of creatinine subsequent to ACR [19]–[21].

Our results were consistent with previous reports in that some studies have demonstrated an association of microalbuminuria with CVD risk factors, including hypertension, hypertriglyceridemia, impaired fasting glucose, and diabetes [22], [23]. It is well known that factors such as hypertension and diabetes are associated with the presence of microalbuminuria [24]–[26], as was also clearly evident from the results of the present study. Older age is a well-known independent risk factor for microalbuminuria and CKD, even in subjects without diabetes or hypertension [24], [26], [27]. Logistic regression analyses in this study indicated that greater age was associated with microalbuminuria and macroalbuminuria. Additionally, we found that older age was associated with decreased eGFR (Table 3). The prevalence of decreased eGFR was much different between subjects aged 19–39 years and those ≥65 years old (0.1% vs. 13.2%, respectively, *P*<0.001). These findings were in agreement with the observation that aging is associated with senescence and interstitial fibrosis and with renal ischemia due to intrarenal arteriosclerosis and cholesterol emboli involvement [28]. In this study, we used a single cut-off point (≥30 mg/g Cr) of albuminuria for men and women because this cut-off point is widely accepted in clinical setting and found that men were less likely to have microalbuminuria compared with women (OR, 0.65; 95% CI, 0.49–0.85, *P* = 0.002). This association was also found in NHANES and AusDiab studies [24], [26], [27]. However, single cut-off point might overestimate the prevalence in women because urinary creatinine excretion is higher in men compared with women [29], [30]. Although single cut-off point is widely accepted in clinical setting, further investigations are required to determine the mechanisms underlying the roles of sex hormones in the development of microalbuminuria and the cut-off point for each sex in practice.

Although the association between hypertriglyceridemia and albuminuria, including microalbuminuria, was not statistically significant in multiple logistic regression analysis, it appeared to be a significant independent predictor of macroalbuminuria in this study. With adjustment for age and sex, the significant positive trend was observed between across categories of albuminuria and all components of metabolic syndrome (waist circumference, TG, hypertension, and fasting plasma glucose; all *P*<0.001). Insulin resistance was suggested to play a role in the development of microalbuminuria by raising glomerular hydrostatic pressure, increasing renal vascular permeability, aggregating glomerular hyperfiltration, and enhancing renal sodium reabsorption [31]. There are several possible mechanisms linking visceral obesity and microalbuminuria, such as systemic inflammation, adipokines, and activation of the sympathetic nervous system and renin–angiotensin system [32]. Although indices of insulin resistance were not measured in this study, hypertriglyceridemia and elevated AST level, which is indicative of non-alcoholic fatty liver disease as another indication of an insulin-resistant clinical phenotype [33], [34], were independently associated with macroalbuminuria and albuminuria, respectively.

Although microalbuminuria has been considered a marker of glomerular injury and an early indicator of progression to renal insufficiency, 64% of the subjects with decreased eGFR had normal albuminuria in the present study. These findings were consistent with the results of the Framingham Heart Study, in which about only one-quarter of subjects with decreased eGFR (<60 ml/min/1.73 m^2^) had microalbuminuria (ACR, at least 30 mg/g Cr) [35], and the Takahata Study, in which 20.5% of Japanese subjects with decreased eGFR had microalbuminuria [36]. Microalbuminuria and decreased GFR were suggested as markers of endothelial dysfunction and renal dysfunction, respectively [37]. However, the mechanisms by which different renal injuries cause glomerular endothelial dysfunction or renal atherosclerosis should be clarified in future studies.

These results of the present study clearly indicated the prevalence of microalbuminuria and its associated factors in Korea. However, this study had some limitations. The first is that a single urine albumin creatinine ratio result was used in this analysis. A single measurement of urine albumin excretion and serum creatinine for calculating ACR and eGFR could result in misleading classifications of albuminuria and CKD stages. However, repeated measurement of these parameters or direct measurement of eGFR is both time consuming and costly and is therefore not feasible for nationwide health examination studies. Additionally, single-point measurement of ACR has been used to predict increased CVD risk [38], [39]. Second, in this study, the proportion of subjects taking antihypertensive agents was 14.9% (95% CI, 13.7–16.3). These subjects may have been taking different antihypertensive agents, which would influence the appearance of microalbuminuria. However, we did not analyze the effects of various antihypertensive agents on the occurrence of microalbuminuria. Finally, this study did not establish a causal relationship because of its cross-sectional nature. Thus, the possibilities of causal relationships remain to be elucidated by prospective observation of the relationships between risk factors outlined in this study and the development of microalbuminuria.

The results of this population-based study indicated the prevalence of microalbuminuria was slightly lower than those in Western population but comparable to those in Asian population. These may suggest ethnic difference although we do not have the data of direct comparison between each ethnic group. However, the presence of microalbuminuria is a matter of concern because this condition is predictive of progressive deterioration of kidney function and is associated with increased risk of CVD events and death. Therefore, identification and management of relevant risk factors in individual with microalbuminuria is an important step to develop targeted preventive efforts in practice because a growing body of evidence suggests that albuminuria is strongly associated with the risk of adverse outcomes even in general populations.
